# Comparison of Extracellular Vesicles from Induced Pluripotent Stem Cell-Derived Brain Cells

**DOI:** 10.3390/ijms25073575

**Published:** 2024-03-22

**Authors:** Gabriela Xavier, Alexander Navarrete Santos, Carla Hartmann, Marcos L. Santoro, Nicole Flegel, Jessica Reinsch, Annika Majer, Toni Ehrhardt, Jenny Pfeifer, Andreas Simm, Thomas Hollemann, Sintia I. Belangero, Dan Rujescu, Matthias Jung

**Affiliations:** 1 LiNC—Laboratory of Integrative Neuroscience, Universidade Federal de São Paulo (UNIFESP), São Paulo CEP 05039-032, Brazil; 2 Genetics Division, Department of Morphology and Genetics, Universidade Federal de São Paulo (UNIFESP), São Paulo CEP 04023-900, Brazil; 3 Centre for Medical Basic Research, Medical Faculty, Martin Luther University Halle-Wittenberg, 06120 Halle (Saale), Germany; 4 Institute for Physiological Chemistry, Medical Faculty, Martin Luther University Halle-Wittenberg, 06114 Halle (Saale), Germany; carla.hartmann@uk-halle.de (C.H.);; 5 Institute for Biochemistry, Friedrich Alexander University Erlangen-Nürnberg, 91054 Erlangen, Germany; nicole.flegel@fau.de; 6 Clinic for Cardiac and Thoracic Surgery, Martin Luther University Halle-Wittenberg, 06120 Halle (Saale), Germany; 7 Department of Psychiatry and Psychotherapy, Medical University of Vienna, 1090 Vienna, Austria

**Keywords:** extracellular vesicles (EVs), induced pluripotent stem cells (iPSCs), neural differentiation, astrocytes, brain capillary endothelial cells (BCECs), schizophrenia, late-onset Alzheimer’s disease (LOAD)

## Abstract

The pathophysiology of many neuropsychiatric disorders is still poorly understood. Identification of biomarkers for these diseases could benefit patients due to better classification and stratification. Exosomes excreted into the circulatory system can cross the blood–brain barrier and carry a cell type-specific set of molecules. Thus, exosomes are a source of potential biomarkers for many diseases, including neuropsychiatric disorders. Here, we investigated exosomal proteins produced from human-induced pluripotent stem cells (iPSCs) and iPSC-derived neural stem cells, neural progenitors, neurons, astrocytes, microglia-like cells, and brain capillary endothelial cells. Of the 31 exosome surface markers analyzed, a subset of biomarkers were significantly enriched in astrocytes (CD29, CD44, and CD49e), microglia-like cells (CD44), and neural stem cells (SSEA4). To identify molecular fingerprints associated with disease, circulating exosomes derived from healthy control (HC) individuals were compared against schizophrenia (SCZ) patients and late-onset Alzheimer’s disease (LOAD) patients. A significant epitope pattern was identified for LOAD (CD1c and CD2) but not for SCZ compared to HC. Thus, analysis of cell type- and disease-specific exosome signatures of iPSC-derived cell cultures may provide a valuable model system to explore proteomic biomarkers for the identification of novel disease profiles.

## 1. Introduction

The pathophysiology of neuropsychiatric disorders is complex and diverse. Diagnoses are justified mainly by structured interviews and therefore mirror the experience of clinicians, emphasizing the need for non-biased biomarkers. Recent technological advances have led to a re-evaluation of extracellular vesicles (EVs), particularly exosomes, as biomarkers for neuropsychiatric disorders [1]. EVs contain constituents of the cell type of origin, including DNA, RNA, lipids, metabolites, and cytosolic and cell surface proteins. In contrast to ectosomes, which pinch off from the surface of the plasma membrane via outward budding (such as microvesicles), exosomes have an endosomal origin [2]. Exosomes are small membranous vesicles (40–160 nm in diameter) that are secreted for cell-to-cell communication and regulate various biological processes in the human brain, including neurogenesis, aging, and disease. Exosomes can carry epitopes on their surface that are specific for the cell type they emerged from. In addition, the lumen of exosomes contains substances that provide an indication of the metabolic processes present in the cell of origin [2]. Together, the molecular composition of exosome epitopes and their lumen contents may provide an indication of a disease-specific state. Because brain-derived exosomes can cross the blood–brain barrier (BBB), ordinary blood samples may be a relatively non-invasive source of potential biomarkers for neuropsychiatric diseases [1].

Cellular models that reflect the native state at the BBB in health and disease would be a powerful tool to better understand cell type-specific exosomal biomarker profiles [3,4]. For this purpose, we utilized an in vitro differentiation model using human-induced pluripotent stem cells (iPSCs) to generate six different brain cell types from healthy control (HC) individuals, schizophrenia (SCZ) patients, and late-onset Alzheimer’s disease (LOAD) patients. Because the contribution of the different cell types to the neuropathology of SCZ and LOAD is different [5,6], we did not analyze all cell types in each disease. We focused our analysis on the most important cell types in each case. The abundance of 31 exosome epitopes from the corresponding iPSCs and iPSC-derived astrocytes, brain capillary endothelial cells (BCECs), microglia-like cells (MGCs), neural stem cells (NSCs), neural progenitor cells (NPCs), and neurons was quantified. Our results suggest that some of the iPSC-derived brain cell types secrete exosomes in a cell type- and/or disease-related manner, which may help support the diagnosis and treatment of patients.

## 2. Results

**Figure 1:**
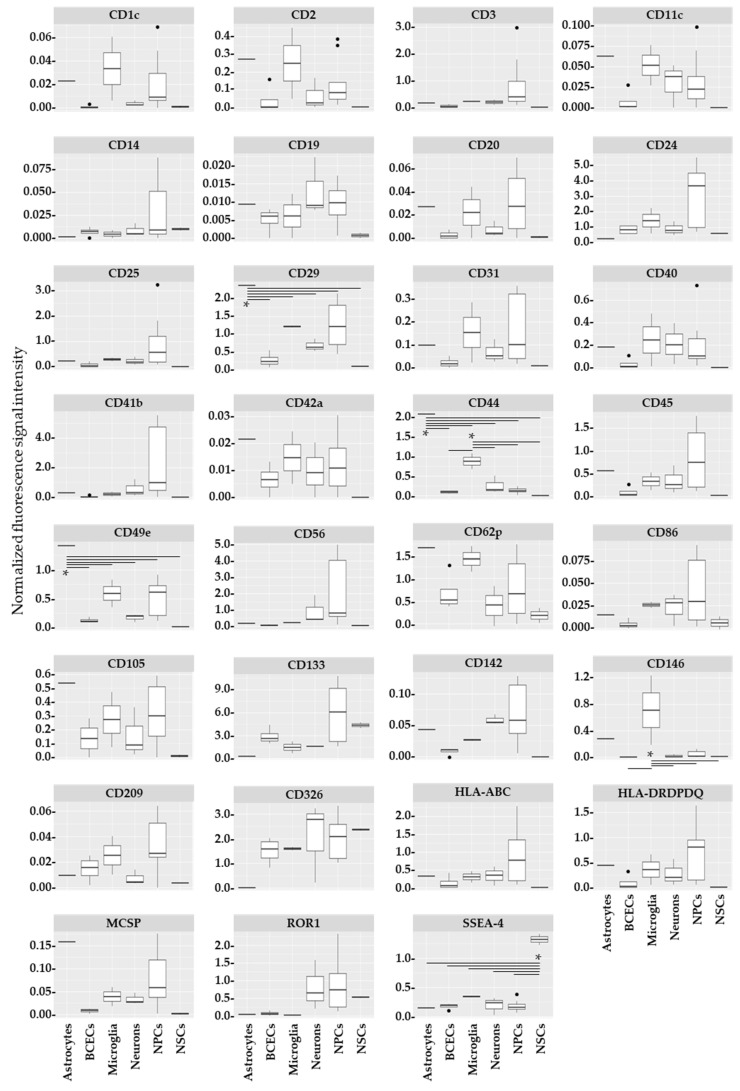
Exosome epitopes related to different cell types in healthy control (HC) individuals. Box plot of normalized fluorescence intensities comparing different HC cell types. Charts are shown for each of the 31 epitopes used for exosome profiling. BCECs: brain capillary endothelial cells; NPCs: neural progenitor cells; NSCs: neural stem cells; ●: outliers; *: *p* < 0.05 after post hoc test (see also Supplementary Table S1). Fluorescence signal intensity unit: MESF.

EVs were harvested from culture media of iPSCs (*n* = 2) and 6 disease-relevant iPSC-derived brain cell types (*n* = 4 astrocytes, *n* = 10 BCECs, *n* = 5 MGCs, *n* = 4 NSCs, *n* = 14 NPCs, and *n* = 8 neuron cells) for the analysis of 31 exosome epitopes (Table 1). None of the epitopes were exclusive to one cell type. Six of the exosome epitopes were significantly altered: CD29 (F = 8.70; *p* = 3.06 × 10^4^), CD44 (F = 33.12; *p* = 3.46 × 10^−8^), CD49e (F = 12.99; *p* = 2.63 × 10^−5^), CD146 (F = 4.01; *p* = 1.82 × 10^2^), MCSP (F = 4.45; *p* = 9.84 × 10^−3^), and SSEA4 (F = 50.99; *p* = 1.22 × 10^−9^) (Table 2). Post hoc analysis showed exosome epitope enrichment for astrocytes (CD29, CD44, and CD49e), microglia (CD44 and CD146), and NSCs (SSEA4) (Figure 1). CD29, CD44, CD49e, and SSEA4 remained significant after adjusting *p* values using Bonferroni correction (Supplementary Table S1).

To identify disease-related signatures, exosome epitopes of EVs from patient cells (both SCZ and LOAD) were compared against cells from HC individuals. Differences were found between the two groups for the epitopes CD14 (U = 121.0; *p* = 0.024), CD133 (U = 174.0; *p* = 0.030), CD146 (U = 115.0; *p* = 0.035), and SSEA4 (U = 177.0; *p* = 0.035), but they were not significant after Bonferroni correction, suggesting that there is no overall discriminating epitope for this comparison (Supplementary Table S2).

**Figure 2:**
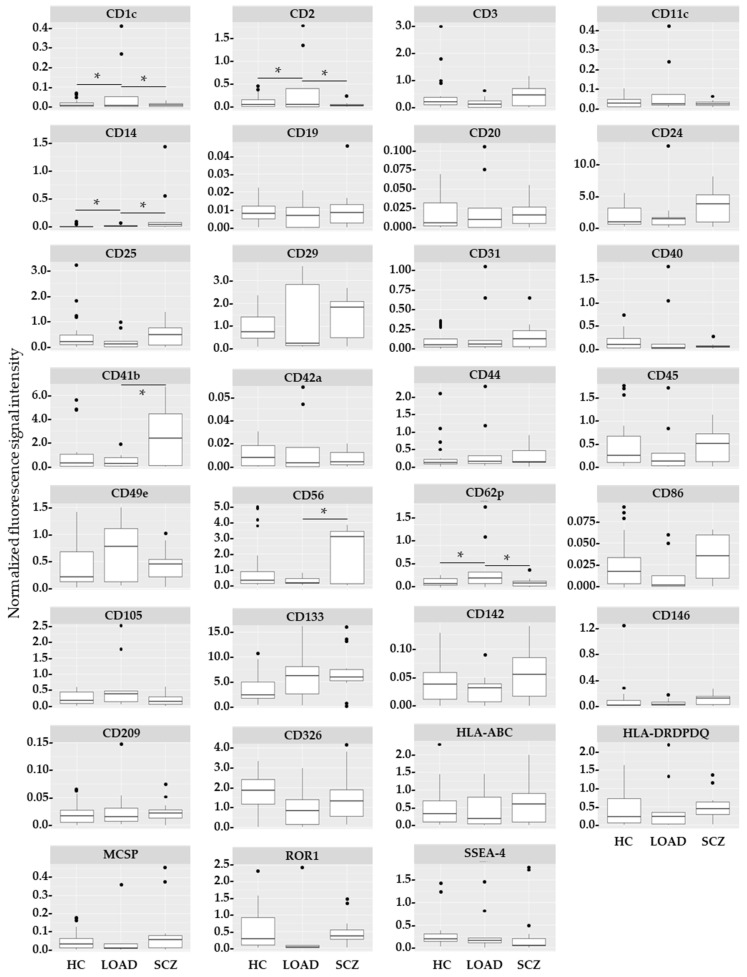
Group-specific exosome epitope measurements. Box plot of normalized fluorescence comparing healthy control, schizophrenia (SCZ), and late-onset Alzheimer’s disease (LOAD) groups. All available cell types of the respective groups were considered for this analysis: HC (astrocytes, BCECs, MGCs, NSCs, NPCs, and neurons), LOAD (astrocytes, BCECs, and MGCs), and SCZ (MGCs, NSCs, NPCs, and neurons). Charts are shown for each of the 31 exosome-identifying epitopes. ●: outliers; *: *p* < 0.05 after post hoc test (see also Supplementary Tables S1 and S3). Fluorescence signal intensity unit: MESF.

**Figure 3:**
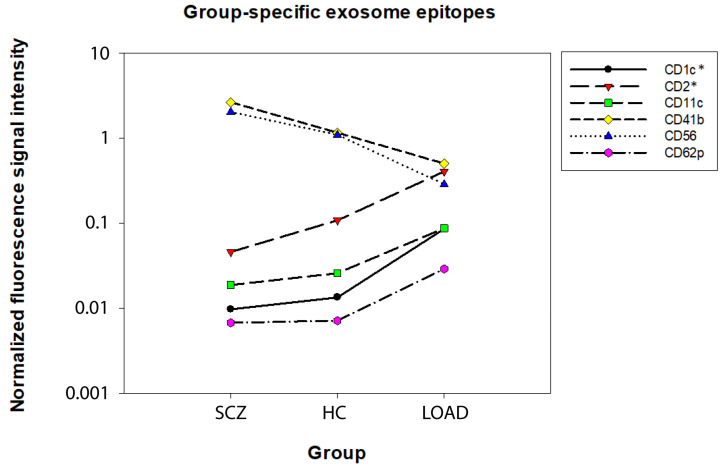
Exosome epitope detection in cells derived from healthy control (HC) individuals. The chart shows flow cytometry measurements for exosome epitopes that have been shown to be significantly regulated among cell types and grouped by their expression. LOAD: late-onset Alzheimer’s disease; SCZ: schizophrenia. *: *p* < 0.05 adjusted *p*-value after Bonferroni correction. Fluorescence signal intensity unit: MESF.

Next, exosome surface epitopes from all available cells in the three groups (HC, SCZ, and LOAD) were compared. Six exosome epitopes of cells from LOAD patients were significantly altered in a post hoc analysis compared to cells from SCZ patients and/or cells from HC individuals: CD1c (F = 8.93; *p* = 9.04 × 10^−4^), CD2 (F = 8.30; *p* = 9.95 × 10^−4^), CD11c (F = 4.60; *p* = 1.67 × 10^−2^), CD41b (F = 3.92; *p* = 2.72 × 10^−2^), CD56 (F = 4.72; *p* = 1.40 × 10^2^), and CD62p (F = 5.82; *p* = 6.36 × 10^−3^). These changes were mainly driven by either epitope enrichment (CD1c, CD2, CD11c, and CD62p) or depletion (CD41b and CD56) in LOAD cells compared to both other conditions (Figure 2). HC showed intermediate values between LOAD and SCZ (Figure 3). Differences observed for CD1c and CD2 remained significant after Bonferroni correction (Supplementary Table S3).

Cell type-specific changes between the HC and SCZ or LOAD groups were also assessed. A comparison between HC and LOAD did not show any significant changes in exosome markers from different cell types. However, 13 exosome epitopes changed in neurons when comparing HC and SCZ: CD3, CD14, CD24, CD25, CD29, CD31, CD41b, CD49e, CD56, CD133, CD142, CD209, and HLA ABC (all U = 0; *p* = 2.50 × 10^−2^). All epitopes lost their significance after Bonferroni correction.

## 3. Discussion

The ability of iPSC models to mimic disease-related pathogenic phenotypes has been previously described in SCZ and LOAD for neuronal [7,8], glial [9], and BBB [10,11] cells. We recently reviewed the potential of iPSC-based BBB models for preclinical drug discovery [3]. In this study, iPSCs from LOAD and SCZ patients as well as from healthy individuals were used to profile EVs from different cell types and to identify exosome epitope signatures associated with specific cell types and diseases. The LOAD and HC iPSCs utilized in the present study have been characterized [12] and were recently used to generate neuronal cells, glial cells, and BCECs [13,14], confirming the applicability and high efficiency of the in vitro differentiation models used.

**Figure 4:**
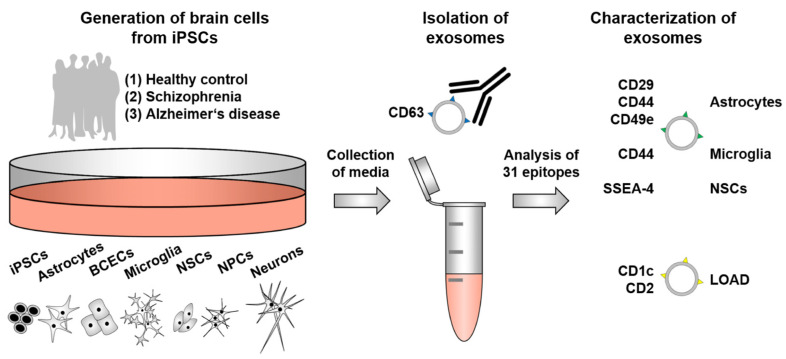
Profiling of exosome epitopes from neuronal cells of healthy controls and patients with neuropsychiatric disorders. Exosomes from different cell types were analyzed: induced pluripotent stem cells (iPSCs) and iPSC-derived neural progenitors, neurons, astrocytes, microglia, and brain capillary endothelial cells (BCECs). By detecting cell type- and disease-specific signatures using in vitro cultures, a model system suitable for further exploration of exosomes was established. LOAD: late-onset Alzheimer’s disease; NSCs: neural stem cells; NPCs: neural progenitor cells.

As hypothesized, distinct exosome epitope profiles were detected in EVs derived from different cell types (Figure 4). Four exosome epitopes were enriched in EVs from astrocytes, microglia, or NSCs. CD29 (integrin beta-1 (ITGB1)) and CD49e (integrin alpha-5 (ITGA5)) were enriched in EVs from human iPSC-derived astrocytes, which corresponds to their crucial role in this cell type in regulating cell adhesion and recognition [15,16]. The multifunctional surface protein CD44 was relatively increased in EVs derived from astrocytes and microglia, which is consistent with its known respective functionalities in migration and activation in these cell types [17]. Stage-specific embryonic antigen 4 (SSEA4) had high detection levels in EVs from NSCs, which is supported by other studies reporting SSEA4 as a marker of human neural progenitors [18]. Differences in expression levels were also observed for melanoma chondroitin sulfate proteoglycan (MCSP; also known as chondroitin sulfate proteoglycan 4 (CSPG4)) in astrocytes and CD146 (cell surface glycoprotein MUC18 (MCAM)) in microglia, but these were not significant following Bonferroni correction. This trend is supported by research showing that CSPG4 expression is present in endothelial cells, neurons, and glial cells and is only altered in astrocytes in the context of disease [19]. A similar pattern holds true for CD146, which is altered when the interaction between endothelial cells and pericytes is disturbed [20].

When comparing exosomal epitopes from healthy and diseased cells (i.e., HC versus SCZ + LOAD), epitopes with differential expression have also been recently described in association with SCZ and LOAD. However, the measured values for CD14, CD133, CD146, and SSEA4 varied widely and lost significance after Bonferroni correction. This result may reflect the different etiologies of these diseases, i.e., while SCZ is a neurodevelopmental disorder, LOAD has a neurodegenerative pathophysiology. CD14 is involved in the recognition of lipopolysaccharides in bacteria and is an important component of the non-specific immune response, especially in immune and endothelial cells but also within the central nervous system. Although CD14 plays a major role in neuroimmunomodulatory processes [21], there is little evidence that it has a crucial role in the pathogenesis of LOAD [22]. In contrast, there are several reports of its involvement in the pathogenesis of SCZ [23,24]. CD133 (prominin-1 (PROM1)) is a marker for specific cells (e.g., neuronal progenitor cells [25,26]), which may explain why significant results were not obtained in this analysis.

The comparisons between HC-SCZ and HC-LOAD only showed significant changes between HC and LOAD. CD1c and CD2 were found to be relatively enriched in LOAD samples. This result is consistent with findings that CD1c and CD2 were elevated in exosomes isolated from the blood plasma of patients suffering from Parkinson’s disease [27], a neurodegenerative disorder with many similarities to LOAD. Most likely, the LOAD-dependent induction of CD1c and CD2 is related to a disturbed immune system.

Results that were only significant before Bonferroni correction concerned epitopes that were already found in glial cells and were also described in connection with neurodegeneration. CD11c (integrin alpha-X, ITGAX) and CD62 have previously been described in the context of Alzheimer’s disease [28,29]. CD41b (integrin alpha IIb (ITGA2B)) and CD56 (neural cell adhesion molecule 1 (NCAM1)) regulate cell adhesion in the brain, and both were elevated in SCZ [30]. Alterations in CD41b have been linked to neuropsychiatric diseases, including upregulation in individuals with autism spectrum disorders [31]. CD56 has been extensively studied in the context of SCZ and other neuropsychiatric disorders. For example, DNA risk variants associated with SCZ have been identified [32,33], and dysregulation of CD56 has been reported in SCZ patients [34].

Comparison of healthy and diseased cell types did not show significant changes in LOAD cells, but significant differences were found for SCZ neurons (CD133 and others), although no result remained significant after Bonferroni correction. Nevertheless, epitopes such as CD133 have recently been described as biomarkers for acute psychosis [35], and therefore larger sample sizes may be necessary to detect significant changes.

Although the flow cytometry-based technique utilized here has only recently become available, it has already found application in many studies on patients [36,37] and is suitable for the analysis of iPSC-derived EVs [38]. The limitations of the current study are the lack of all cell types in all groups and the limited number of biological and technical replicates. Also, the small sample size due to the number of available iPSC cell lines, especially for LOAD and SCZ, resulted in a limited statistical basis and requires cautious interpretation of the results.

In summary, in our model, we found that specific exosome epitopes significantly accumulated in certain cell types in LOAD (compared to HC) or in SCZ neurons (compared to healthy neurons). However, only the cell type-specific results for astrocytes, microglia, and NSCs (CD29, CD44, CD49e, CD146, and SSEA4) and the disease-specific data for LOAD cells (CD1c and CD2) were significant after Bonferroni correction. These findings suggest that larger sample sizes are necessary for future studies.

## 4. Materials and Methods

### 4.1. Origin and Characterization of iPSCs

Control cell lines WISCi004 B and WAi001-A were purchased from the WiCell Research Institute (Madison, WI, USA). Human iPSC lines from two SCZ patients (MLUi001-M and MLUi002-G), one LOAD patient (MLUi007-H/J), and one HC individual (MLUi009-A) were generated in-house from B-lymphoblastoid cells and registered in a global registry for human pluripotent stem cell lines (https://hpscreg.eu, accessed on 20 March 2024). For the generation of human iPSCs, subjects’ blood was collected, and peripheral blood mononuclear cells were isolated for immortalization by Epstein–Barr virus infection. Obtained B-lymphoblastoid cell lines were then used to generate iPSCs by electroporation with episomal vectors, as recently described [12]. The somatic donor cells and the resulting iPSCs were characterized extensively, the latter for their pluripotency characteristics, differentiation capacity, and genomic integrity. Normal karyotype was not verified for MLUi007 H possessing karyotype XX46 tr (1,13) tr (2,11).

SCZ iPSCs were obtained from two SCZ patients (both male, ages 22 and 37) of European descent and were selected from PAGES (Phenomics and Genomics Sample) [39]. DSM-IV and ICD-10 criteria were fulfilled for SCZ through the collection of medical and psychiatric histories, including the Structured Clinical Interview for DSM IV (SCID), to evaluate lifetime Axis I and II diagnoses [40]. Furthermore, both patients carried a heterozygous deletion (copy number variants) in the NRXN1 gene, representing a high risk factor for SCZ [41]. LOAD iPSCs were derived from a European LOAD patient (female, age 76) who was recruited according to NINCDS-ADRDA criteria [42] at the outpatient clinic of the Department of Psychiatry, University of Munich, Germany. The patient was diagnosed according to the diagnostic and statistical manual of mental disorders (DSM IV) and carried a homozygous APOE ε4 genotype (APOE4/4). A European person (female, age 64) was selected as a matched HC individual. There was an absence of central neurological disease and psychotic disorders in the control subject, including first-degree relatives, using SCID I and II [43] and the Family History Assessment Module [44]. The HC individual carried a homozygous APOE ε3 genotype (APOE3/3).

### 4.2. Cultivation of Human iPSCs

Cells were grown at 37 °C in a humidified environment under hypoxic conditions (5% O_2_, 5% CO_2_, and 90% N_2_). Cells were cultured in mTeSR^TM^1 (Stemcell Technologies, Cologne, Germany) on Matrigel^TM^-coated 6-well plates (VWR, Darmstadt, Germany; 0.083 mg/well) in 1 mL Knockout^TM^ DMEM (Thermo Fisher Scientific, Bremen, Germany). For passaging, cells were treated with 1 mg/mL collagenase IV (Thermo Fisher Scientific) in Knockout DMEM for 5–7 min at 37 °C, followed by rinsing once in Knockout DMEM, and subsequently mechanical dissociation and seeding at a 1:10–1:100 split ratio with daily medium replacement. 

### 4.3. Generation of NSCs, NPCs, and Neurons

Generation of neurons via NSCs and NPCs was performed as previously described [14] with minor modifications. Human iPSCs were dissociated with Accutase^TM^ and seeded onto Matrigel-coated plates at a cell density of 15–25 × 10^3^ cells/cm^2^ in mTeSR1 supplemented with 10 µM protein kinase inhibitor HA 100 (Santa Cruz Biotechnology, Heidelberg, Germany). When cells reached 15–20% confluence, the medium was switched to StemDiff^TM^ Neural Induction Medium (NIM; Stemcell Technologies). NIM medium was changed daily until the generation of primitive NSCs on day 7. Subsequently, the NSCs were dissociated with Accutase and seeded at a cell density of 50 × 10^3^ cells/cm^2^ on Matrigel-coated plates. The cells were seeded in terminal differentiation medium I consisting of 25% DMEM, 25% Ham’s F12 nutrient mixture, and 50% Neurobasal^TM^ with 1X N-2 supplement, 1X B 27^TM^ supplement minus vitamin A (all Thermo Fisher Scientific), 0.3 µg/mL cyclic adenosine monophosphate (cAMP; Stemcell Technologies), 1 µM cyclopamine-KAAD (Merck, Darmstadt, Germany), and 1 ng/mL murine Wnt-3a (Peprotech, Hamburg, Germany) to differentiate into NPCs. Around day 40, cells were detached with Accutase and reseeded to avoid the formation of clumps. Cell culture dishes were first coated with 0.1 mg/mL poly-L-ornithine solution and then with 5 µL/mL laminin (both Merck). Differentiation into mature neurons was completed around day 60. The medium was changed every second day. The medium was collected at day 60 for exosome isolation.

### 4.4. Generation of Astrocytes

NSCs were generated as outlined earlier and then differentiated into astrocytes based on a previously published protocol [45] with minor modifications as recently described [14]. NSCs were cultured in 25% DMEM, 25% Ham’s F12 nutrient mix, and 50% Neurobasal with 0.5X N-2 supplement, 0.5X B 27 supplement minus vitamin A, 2 mM GlutaMAX^TM^ (Thermo Fisher Scientific), 10 ng/mL hbFGF (also known as FGF2), and 10 ng/mL EGF (both Peprotech) for at least two days. To initiate astrocyte differentiation, the medium was supplemented with 1X N-2 supplement, 4% fetal calf serum (Lonza, Cologne, Germany), and 10 ng/mL CNTF (Peprotech). At day 16, CNTF was replaced by 0.5 mM dibutyryl cyclic adenosine monophosphate (dibutyryl cAMP; Sigma-Aldrich, Taufkirchen, Germany). At day 23, the medium was supplemented with 1X N-2 supplement and 4% fetal calf serum. At day 30, cells were passaged on T25 flasks coated with 10 µg/mL poly-L-lysine and cultured in AC medium (both Pelo Biotech, Planegg, Germany). The medium was changed every second day. The medium was collected at day 40 for exosome isolation.

### 4.5. Generation of MGCs

MGCs were generated based on previously published protocols [46,47] with minor modifications as recently described [14]. Human iPSCs were passaged one day before differentiation and seeded in small colonies. Cells were kept under hypoxic conditions (5% O_2_, 5% CO_2_, and 90% N_2_) until day 9. Differentiation into microglia progenitors was induced by cultivation in mTeSR1 medium supplemented with 80 ng/mL BMP4. On day 2, 10 µM protein kinase inhibitor HA100 (Santa Cruz) was added to the differentiation medium. At day 4, cells were cultivated in StemPro^TM^-34 SFM (Thermo Fisher Scientific) supplemented with 2 mM GlutaMAX, 80 ng/mL VEGFB, 20 ng/mL FGF2, and 100 ng/mL SCF. At day 6, growth factors were changed to 60 ng/mL SCF, 60 ng/mL IL-3, 50 ng/mL M-CSF, 50 ng/mL FLT3-L, and 5 ng/mL TPO. At day 12, cell growth factors were changed to 50 ng/mL FLT3-L, 50 ng/mL GM-CSF, and 50 ng/mL M-CSF (all growth factors from Peprotech). The medium was changed every third day until day 24. Between days 20 and 24, detaching hematopoietic precursors were collected from the supernatant, seeded on 0.1% gelatin-coated plates, and cultivated for 24 h under hypoxic conditions to improve cell attachment. At day 25, cells were cultured under normoxic conditions in serum-free neuroglial differentiation (NGD) medium [48] by changing the medium every other day. NGD consisted of neurobasal with 1X B-27 supplements without vitamin A, 1X N-2 supplement, 2 mM GlutaMAX, 10 ng/mL biotin (PanReac AppliChem, Darmstadt, Germany), 1 mM sodium pyruvate, 0.02% lactic acid (85.0% syrup), 2 mg/mL lipidated bovine serum albumin, 2.5 mg/L L-ascorbic acid (all Merck), 50 mM NaCl (Carl Roth, Karlsruhe, Germany), 100 ng/mL IL-34, 10 ng/mL M-CSF, and 5 ng/mL GM-CSF. The medium was collected at day 60 for exosome isolation.

### 4.6. Generation of BCECs

BCECs were generated based on previously published protocols [49,50] with minor modifications as recently described [14]. Then, 2–5 days before differentiation, human iPSCs were passaged with Accutase and seeded in mTeSR1 supplemented with 10 µM HA-100 (only for seeding) on Matrigel-coated 6-well plates with a defined number of cells (7.5–12.5 × 10^3^ cells/cm^2^). To initiate differentiation, cells were treated with unconditioned medium containing 78.5% DMEM/F12 without glutamine, 20% Knockout serum replacement, 1 mM GlutaMAX, 1% non-essential amino acids (all Thermo Fisher Scientific), and 0.1 mM 2 mercaptoethanol (Merck). The cells were refreshed every day until day 5. At day 6, the medium was changed to EC medium containing 99% human endothelial serum-free medium and 1% platelet-poor plasma-derived bovine serum (Alfa Aesar^TM^, Thermo Fisher Scientific) supplemented with 20 ng/mL FGF2 and 10 µM all-trans retinoic acid (Sigma-Aldrich). At day 8, 1 × 10^6^ cells/cm^2^ were seeded onto collagen IV/fibronectin-coated inserts (0.4 µm pore size; Greiner Bio One, Frickenhausen, Germany) with 1 mg/mL collagen IV and 0.5 mg/mL fibronectin (both Merck) in deionized water. At day 9, cells were grown without FGF2 and all-trans retinoic acid. The medium was collected on day 11 for exosome isolation.

### 4.7. Isolation of EVs and Analysis of Exosome Epitopes

The media for exosome isolation were collected after 48 h of cell culture. The supernatants were frozen in liquid nitrogen and stored at −80 °C. Exosomes were extracted from 0.4 mL (BCECs) and 2.0 mL (iPSCs, NSCs, NPCs, neurons, astrocytes, and MGCs) of medium. Samples were concentrated using three centrifugation steps: 300× *g* for 10 min, 2000× *g* for 30 min, and 10,000× *g* for 45 min. EVs were isolated using the Exosome Isolation Kit CD63 (Miltenyi Biotec, Bergisch Gladbach, Germany), and the protein content of samples was measured using the BCA Protein Assay micro Kit (Thermo Fisher Scientific). Isolated exosomes were snap-frozen and stored at −80 °C.

Samples were stained for exosome epitopes using the MACS Plex Exosome Kit (Miltenyi Biotec) and quantified using a BD LSR Fortessa (BD Biosciences, Heidelberg, Germany) instrument to detect 39 exosome epitopes according to the manufacturer’s protocol. Fluorescence signals were gated and processed according to the manufacturer’s instructions (Supplementary Figure S1). Data were corrected by subtraction of measurements from sample-free runs. Epitope CD63 was used for exosome isolation and was therefore excluded from the analysis. Because CD9 and CD81, like CD63, also represent abundant EV markers, the data for each sample tested were normalized to measurements for CD9 and CD81 as internal controls. The data were also normalized to protein content. Epitopes with less than 50% of valid measurements (CD4, CD8, and CD69) were excluded. The negative controls, mlG1 and recombinant antibodies (REA), were shown to be negative and were not included in further analyses. After subtracting the five markers for quality control and the three less expressed epitopes, 31 markers were included in the statistical analyses.

### 4.8. Statistical Analysis

Statistical analysis was performed using SPSS (PASW Statistics 18), and graphics were generated with R-Studio 1.3.1093 and R 4.0.3 [51]. To verify cell type-specific differences in epitope compositions, an ANOVA with the Tukey HSD post hoc test was used to compare the measurements of 31 exosome epitopes among the six cell types derived from HC cells. The Mann–Whitney U test evaluated differences in epitope measurements independent of cell type between HC and the combined patient group (SCZ + LOAD). Differences in epitope measurements between HC, SCZ, and LOAD were calculated by ANOVA with the Tukey HSD post hoc test. Finally, the Mann–Whitney U test was performed to compare cell type-specific epitope measurements between HC-, SCZ-, and LOAD-specific cell types. Bonferroni correction was performed for multiple testing of 31 epitopes. For further details, refer to the Supplementary Methods.

## 5. Conclusions

Analysis of exosome epitopes in HC- and patient-specific brain cell types suggests that certain exosomes are enriched or depleted during the disease and that these exosomes originate from specific brain cell types. Further analysis of their content therefore seems promising to better understand diseases and improve therapies.

## Figures and Tables

**Table 1:** Samples from iPSC-derived cell types.

Group		HC	LOAD	SCZ
Cell lines		MLUi009-A WISCi004-B WAi001-A	MLUi007-J MLUi007-H	MLUi001-M MLUi002-G
Cell type	Astrocytes	2	2	-
	BCECs	4	6	-
	MGCs	2	1	2
	NSCs	2	-	2
	NPCs	10	-	4
	Neurons	3	-	5
	Total	23	9	15

-: no sample analyzed; BCECs: brain capillary endothelial cells; HC: healthy control; LOAD: late-onset Alzheimer’s disease; MGCs: microglia-like cells; NSCs: neural stem cells; NPCs: neural progenitor cells; SCZ: schizophrenia.

**Table 2:** Enriched epitopes on exosomes.

Cell Type	Single Cell Type vs. Any Other in HC	LOAD vs. HC, SCZ
Astrocytes	↑CD29 *, ↑CD44 *, ↑CD49e *, ↑MCSP ^1^	↑CD1c * ↑CD2 * ↑CD11c ↓CD41b ^2^ ↓CD56 ^2^ ↑CD62p
BCECs	n.s.	
Microglia	↑CD44 *, ↑CD146	
Neurons	n.s.	
NPCs	n.s.	
NSCs	↑SSEA4 *	

^1^: not significant for Tukey HSD; ^2^: LOAD vs. SCZ only; ↑↓: significant exosome epitope enrichment or reduction shown by ANOVA and post hoc test by Tukey HSD or Mann–Whitney U test for non-normally distributed samples in the depicted cell type compared to any of the other six (for astrocytes and NSCs) or five (for microglia) cell types; *: *p* < 0.05 adjusted *p*-value after Bonferroni correction; BCECs: brain capillary endothelial cells; HC: healthy control; LOAD: late-onset Alzheimer’s disease; NPCs: neural progenitor cells; n.s.: not significant; NSCs: neural stem cells; SCZ: schizophrenia.

## Data Availability

The authors confirm that the data supporting the findings of this study are available within the article and its Supplementary Materials. Raw data supporting the findings of this study are available from the corresponding author upon request.

## Supplementary Materials

The following supporting information can be downloaded at https://www.mdpi.com/article/10.3390/ijms25073575/s1.
