# Supplementary material for: Comparison of Extracellular Vesicles from Induced Pluripotent Stem Cell-Derived Brain Cells
Source: Int J Mol Sci. 2024 Mar 22;25(7):3575. doi: 10.3390/ijms25073575 (PMC11011287; doi:10.3390/ijms25073575)
Supplement: Supplementary file 1 [file ijms-25-03575-s001.zip › ijms-2893776-supplementary.pdf]

Supplementary Figure S1

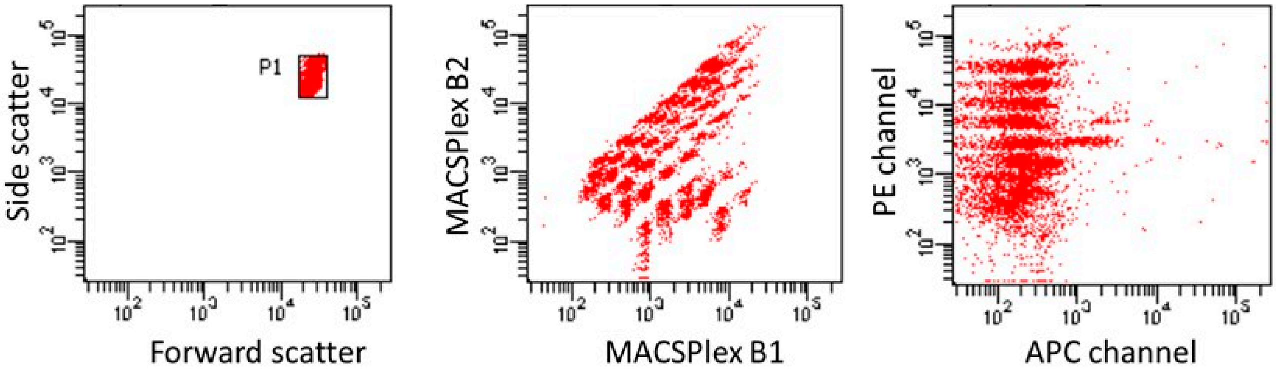

Supplementary Figure S1: Raw data of flow cytometric analysis. Left: gating results using forward and sideward scatter according to bead size are cropped in P1. Middle: dot plot showing the discrimination of differently labeled bead populations of P1. Right: dot plot showing captured bead signals in allophycocyanin (APC) and phycoerythrin (PE) channels for visualization of signal intensities of the single bead populations.

Supplementary Table S1

Supplementary Table S1: Cell type-specific epitope enrichment in HC								
**Marker / encoded by**	**ANOVA**			**Tukey HSD**				
	**df**	**F**	**p-value (adj. p-value)**	**Cell Type I**	**Mean (Stdev)**	**Cell Type II**	**Mean (Stdev)**	**p-value**
**CD29** / ITGB1	5	8.70	3.06E-04 (8.87E-03)	Astrocytes	3.571 (1.71)	BCEC	0.303 (0.20)	1.88E-04
						Microglia	1.227 (0.04)	1.82E-02
						Neurons	0.701 (0.17)	1.36E-03
						NPCs	1.258 (0.64)	2.27E-03
						NSCs	0.133 (0.02)	5.34E-04
**CD44** / CD44	5	33.12	3.46E-08 (1.00E-06)	Astrocytes	1.703 (0.55)	BCEC	0.094 (0.04)	1.05E-07
						Microglia	0.883 (0.29)	2.82E-03
						Neurons	0.259 (0.22)	1.07E-06
						NPCs	0.134 (0.07)	2.88E-08
						NSCs	0.006 (0.00)	3.91E-07
				Microglia	0.883 (0.29)	BCEC	0.094 (0.04)	9.79E-04
						Neurons	0.259 (0.22)	1.35E-02
						NPCs	0.134 (0.07)	5.18E-04
						NSCs	0.006 (0.00)	1.44E-03
**CD49e** / ITGA5	5	12.99	2.63E-05 (7.62E-04)	Astrocytes	1.840 (0.60)	BCEC	0.129 (0.04)	2.02E-05
						Microglia	0.595 (0.34)	3.49E-03
						Neurons	0.176 (0.07)	5.56E-05
						NPCs	0.524 (0.30)	1.30E-04
						NSCs	0.020 (0.01)	5.69E-05
**CD146** / MCAM	5	4.01	1.82E-02 (5.28E-01)	Microglia	0.712 (0.74)	BCEC	0.007 (0.00)	3.66E-02
						Neurons	0.027 (0.03)	4.39E-02
						NPCs	0.046 (0.05)	9.18E-03
						NSCs	0.011 (0.00)	3.82E-02
**MCSP** / CSPG4	5	4.45	9.84E-03 (2.85E-01)					
**SSEA4**	5	50.99	1.22E-09 (3.55E-08)	NSCs	1.323 (0.14)	Astrocytes	0.219 (0.09)	2.46E-08
						BCEC	0.177 (0.05)	1.52E-09
						Microglia	0.347 (0.02)	1.53E-07
						Neurons	0.196 (0.15)	4.43E-09
						NPCs	0.185 (0.09)	2.91E-10
df: degrees of freedom; F: ANOVA test value; adj. p-value: p-values were adjusted for 31 comparisons using Bonferroni correction; HC: healthy control; Stdev: standard deviation; Tukey's HSD: Tukey’s honestly significant difference test alias Tukey’s range test								

Supplementary Table S2

**Supplementary Table S2: Disease-specific epitope enrichment: Comparison of HC with the combined patient group (SCZ + LOAD)**				
**Marker / encoded by**	**U**	**Z**	**p-value**	**Adj. p-value**
**CD14** / CD14	121.000	-2.259	0.024	0.69
**CD133** / PROM1	174.000	-2.171	0.030	0.87
**CD146** / MCAM	115.000	-2.107	0.035	1.00
**SSEA4**	177.000	-2.107	0.035	1.00

U: Mann-Whitney U test value; Adj. p-value: p-values adjusted for 31 comparisons using Bonferroni correction; HC: healthy control. SCZ: schizophrenia. LOAD: late-onset Alzheimer’s disease.

Supplementary Table S3

Supplementary Table S3: Disease-specific epitope enrichment by comparison of HC, SCZ, and LOAD.								
**Marker / encoded by**	**ANOVA**			**Tukey HSD**				
	**df**	**F**	**p-value (adj. p-value)**	**Group I**	**Mean (Stdev)**	**Group II**	**Mean (Stdev)**	**p-value**
**CD1c** / CD1C	2	8.93	9.04E-04 (2.62E-02)	LOAD	0.155 (0.18)	HC	0.017 (0.02)	9.92E-04
						SCZ	0.015 (0.01)	1.90E-03
**CD2** / CD2	2	8.30	9.95E-04 (2.89E-02)	LOAD	0.610 (0.77)	HC	0.113 (0.14)	2.06E-03
						SCZ	0.049 (0.06)	1.03E-03
**CD11c** / ITGAX	2	4.60	1.67E-02 (4.83E-01)	LOAD	0.112 (0.16)	HC	0.033 (0.03)	3.42E-02
						SCZ	0.020 (0.01)	1.61E-02
**CD41b** / ITGA2B	2	3.92	2.72E-02 (7.89E-01)	LOAD	0.565 (0.65)	HC	1.158 (1.83)	7.37E-01[1]
						SCZ	2.647 (2.45)	4.64E-02
**CD56** / NCAM1	2	4.72	1.40E-02 (4.07E-01)	LOAD	0.286 (0.27)	HC	1.092 (1.65)	3.63E-01[1]
						SCZ	2.178 (1.64)	1.32E-02
**CD62p** / SELP	2	5.82	6.36E-03 (1.84E-01)	LOAD	0.033 (0.04)	HC	0.008 (0.01)	7.11E-03
						SCZ	0.008 (0.01)	1.53E-02

df: degrees of freedom; F: ANOVA test value; adj. p-value: p-values were adjusted for 31 comparisons using Bonferroni correction; HC: healthy control; SCZ: schizophrenia; LOAD: late-onset Alzheimer's disease; Stdev: standard deviation; [1]: no significant change.
